# Effect of *Helicobacter pylori* Eradication Treatment on Metachronous Gastric Neoplasm Prevention Following Endoscopic Submucosal Dissection for Gastric Adenoma

**DOI:** 10.3390/jcm12041512

**Published:** 2023-02-14

**Authors:** Choong-Kyun Noh, Eunyoung Lee, Bumhee Park, Sun Gyo Lim, Sung Jae Shin, Kee Myung Lee, Gil Ho Lee

**Affiliations:** 1 Department of Gastroenterology, Ajou University School of Medicine, Suwon 16499, Republic of Korea; 2 Department of Biomedical Informatics, Ajou University School of Medicine, Suwon 16499, Republic of Korea; 3 Office of Biostatistics, Ajou Research Institute for Innovative Medicine, Ajou University Medical Center, Suwon 16499, Republic of Korea

**Keywords:** *Helicobacter pylori*, gastric adenoma, endoscopic submucosal dissection, metachronous gastric neoplasm, eradication treatment

## Abstract

The long-term effect of *Helicobacter pylori* eradication on metachronous gastric neoplasm prevention after endoscopic submucosal dissection (ESD) of gastric adenoma is unclear. This study included patients with confirmed *H. pylori* infection after ESD with curative resection for gastric adenoma. Patients were divided based on the success of *H. pylori* eradication treatment into two groups: eradication and non-eradication. Patients with any newly detected lesion within 1 year after ESD and recurrence at the ESD site were excluded from the analysis. Further, 1:1 propensity score matching was also performed to eliminate baseline differences between the two groups. *H. pylori* eradication treatment was administered to 673 patients after ESD (163 in the successful eradication group and 510 in the non-eradication group). During the median follow-up periods of 25 and 39 months in the eradication and non-eradication groups, metachronous gastric neoplasm was identified in 6 (3.7%) and 22 patients (4.3%), respectively. Adjusted Cox analysis revealed that *H. pylori* eradication was not associated with increased risk of metachronous gastric neoplasm after ESD. Kaplan–Meier analysis in the matched population yielded similar findings (*p* = 0.546). *H. pylori* eradication treatment was not associated with metachronous gastric neoplasm after ESD with curative resection for gastric adenoma.

## 1. Introduction

*Helicobacter pylori* is a strong risk factor in gastric cancer [1,2]; hence, eradication treatment for *H. pylori* may prevent gastric cancer [3,4,5,6]. In general, patients with gastric cancer experience precancerous changes in the gastric mucosa, including atrophic gastritis and intestinal metaplasia [7]. *H. pylori* eradication can prevent progression to advanced precancerous lesions in a gastric carcinogenesis model with atrophic gastritis and intestinal metaplasia. Thus, eradication treatment for *H. pylori* is recommended for gastric cancer prevention [8,9,10].

In contrast, the “point of no return” theory explains that gastric cancer might develop after *H. pylori* eradication treatment [11,12]. This theory questions the role of *H. pylori* eradication treatment when histological changes in the gastric mucosa, such as atrophic gastritis and intestinal metaplasia, are already evident. However, studies involving patients with atrophic gastritis and intestinal metaplasia demonstrated that *H. pylori* eradication treatment reduced the incidence of gastric cancer [13]. Similarly, a meta-analysis study reported an improvement in atrophy after eradication treatment [14].

Gastric adenomas are precancerous lesions that can be eradicated with endoscopic submucosal dissection (ESD) [15,16,17]. *H. pylori* eradication treatment lowers the incidence of metachronous gastric cancer in patients with early gastric cancer [18]; however, the effects of eradication treatment after gastric adenoma resection are controversial. Studies on the incidence of metachronous gastric neoplasm after endoscopic resection for gastric adenoma are lacking, and related retrospective studies have shown contradictory findings [19,20,21]. In the latest Maastricht VI report, *H. pylori* eradication treatment after the resection of early gastric cancer is highly recommended; however, the recommendations are unclear for gastric adenomas [10]. Therefore, this study aimed to evaluate whether the success of *H. pylori* eradication treatment influences metachronous gastric neoplasm in patients with gastric adenomas after ESD with curative resection using real-world data collected over 15 years at a single center.

## 2. Materials and Methods

### 2.1. Study Design and Patients

This retrospective, single-center study included patients who underwent ESD for gastric adenoma at Ajou University Hospital (Suwon, Republic of Korea) between 1 January 2005 and 31 December 2020. Exclusion criteria involved patients who met the following conditions: (1) previous history of gastric adenoma or cancer, (2) previous history of other malignancies and/or multiple gastric lesions in the initial workup, (3) patients who were not confirmed to be infected with *H. pylori* in the initial workup, (4) patients who tried *H. pylori* eradication treatment but successful eradication was not confirmed, and (5) incomplete resection. Incomplete resection was defined when one of the following conditions was not satisfied: En-bloc resection (single-piece specimen) and a negative resection margin (both lateral and deep margins). The enrolled patients were assigned to eradication and non-eradication groups based on the *H. pylori* eradication treatment results. The Ajou University Hospital Institutional Review Board approved protocol of this study (approval number: AJIRB-BMR-MDB-21-650). Because this study was a retrospective study, the process of obtaining informed consent was waived.

### 2.2. Endoscopic Submucosal Dissection

Five highly experienced expert endoscopists (K.M.L., S.J.S., S.G.L., C.K.N., and G.H.L.) conducted ESD at our center. Before resecting the lesions, the entire stomach was inspected for additional lesions, and the lesion margins were evaluated. After observing all lesions with white light endoscopy and narrow-band imaging, an indigo carmine solution was applied to establish accurate margins. Subsequently, a needle knife (Dual knife; Olympus, Tokyo, Japan) was used to mark 5 mm outside the lesion margin. To reduce bleeding during incision and submucosal dissection, epinephrine mixed with saline was injected into the submucosal layer; therefore, the lesion was sufficiently lifted from the proper muscle layer. After inducing a circumferential incision 5 mm from the marked site, a needle or an insulated-tip knife (IT knife; Olympus, Tokyo, Japan) was used to dissect the submucosal layer and separate the lesion from the proper muscle layer. The specimen was removed from the body, pinned to a plate, and immersed in a 10% buffered formalin solution. Subsequently, the specimen was forwarded to the pathology department for histopathologic confirmation.

### 2.3. Tumor Evaluation and Variable Definition

Tumor characteristics were evaluated through endoscopic and pathologic findings. The lesion size was determined by measuring the long diameter of the tumor with an endoscopic ruler, and the specimen size was measured after plate fixation. Gross morphology was divided into elevated, flat, or depressed according to the dominant type [22]. We categorized tumor location into three parts of the stomach based on the longitudinal axis (upper, middle, and lower third). During histopathologic confirmation, tumor size, gastric adenoma histologic type, and presence of atrophy and intestinal metaplasia were also assessed. According to the revised Vienna classification, the histologic type of gastric adenoma was divided into low-grade and high-grade dysplasia [23]. The presence of tumor cells in the lateral and deep resection margin was also assessed. The histological evaluation confirmed the atrophic condition and the existence of intestinal metaplasia. All enrolled cases were re-evaluated by two expert pathologists (Jin Roh and Seokhwi Kim). Additionally, we collected the following baseline information: smoking and alcohol consumption status, family history of gastric cancer, and the American Society of Anesthesiologists (ASA) physical status (1 or 2). Metachronous gastric neoplasm was defined as a new lesion, including adenoma or cancer, detected in a location different from the ESD site more than 1 year after endoscopic resection, because we excluded local recurrence, residual disease (recurrence at the ESD site within 1 year), and synchronous lesions (new lesions, including adenoma or cancer, detected in a location different from ESD site, within 1 year after endoscopic resection).

### 2.4. Confirmation of H. pylori Infection and Eradication Treatment

*H. pylori* infection was confirmed using a urea breath test, a rapid urease test, or pathologic evaluation (hematoxylin and eosin and Wright–Giemsa stain) during the initial workup. The success of the eradication treatment was defined as a negative result of rapid urease test or urea breath test after three or four weeks after standard triple therapy (standard dose of proton pump inhibitor, amoxicillin at 1 g, and clarithromycin at 500 mg twice daily for 7 or 14 days) [24].

### 2.5. Follow-Up Schedules after ESD

Definite surveillance guideline after ESD with curative resection in patients with gastric adenoma does not exist. According to our center protocol, we performed follow-up endoscopies on all patients 3, 6, 12, 18, and 24 months after ESD and annually. Additionally, a biopsy was performed at the ESD sites at each follow-up examination, and an additional biopsy was performed at the operator’s discretion when a new lesion was suspected.

### 2.6. Statistical Analysis

Categorical variables were analyzed using Pearson chi-square or Fisher’s exact test, while continuous variables were analyzed using independent t-test or Wilcoxon rank sum test. Log-rank test was used for the evaluation of differences in disease-free probability between the two groups. The Kaplan–Meier method was used for plotting survival curves for gastric adenoma, cancer, and neoplasm. Hazard ratios (HRs) were estimated using the Cox proportional hazard model to assess the incidence risk of metachronous gastric neoplasm. Propensity score matching was performed to reduce selection bias in this retrospective study. The propensity score was estimated with 11 matching variables, including age, sex, pathology, chronic atrophic gastritis, intestinal metaplasia, lesion size, tumor location, gross morphology, familial history of gastric cancer, smoking, and ASA physical status classification system score. Using these scores, the eradication and non-eradication groups of *H. pylori* infection were matched in a 1:1 ratio. Standardized mean differences were calculated to measure covariate balance before and after propensity score matching. All statistical analyses were performed using R software, version 3.6.2 (R Project for Statistical Computing), and SAS software, version 9.4 (SAS Institute Inc., Cary, NC, USA). Statistical significance was defined as two-sided *p*-values < 0.05.

## 3. Results

### 3.1. Baseline Characteristics of the Eradication and Non-Eradication Groups

**Figure 1:**
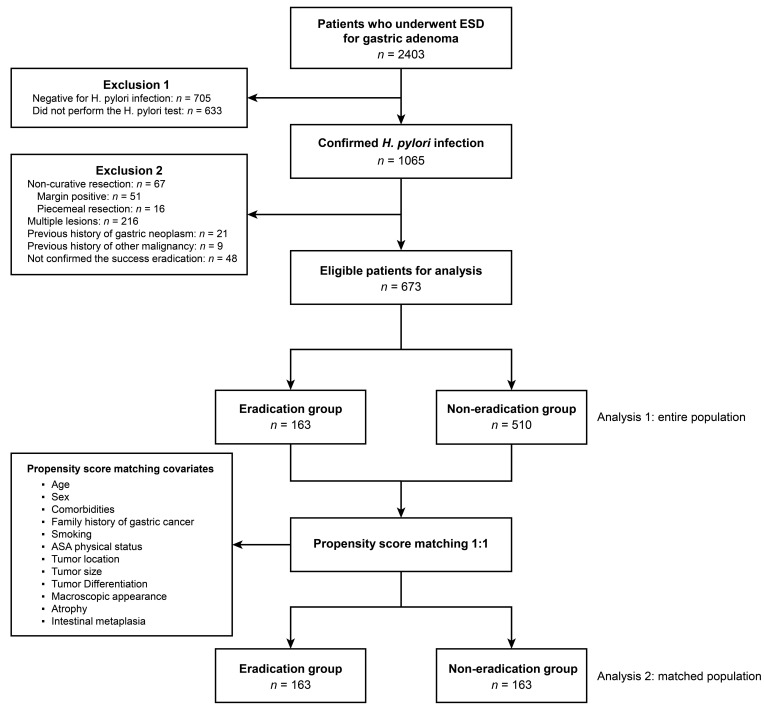
Flow diagram of study population. Abbreviations: ASA, American Society of Anesthesiologists; ESD, endoscopic submucosal dissection; *H. pylori*, *Helicobacter pylori*.

Of the 2403 patients who underwent ESD for gastric adenoma, 1338 patients who tested negative for *H. pylori* infection or who were not tested for *H. pylori* infection were excluded. Among 1065 patients with confirmed *H. pylori* infection, 48 patients were excluded because successful eradication was not confirmed after *H. pylori* eradication treatment. In addition, 67 patients were excluded due to incomplete resection, and 216 patients were excluded due to multiple lesions during the initial workup. A total of 30 patients were excluded because of previous history of gastric neoplasm or other malignancies. Hence, 163 and 510 patients were assigned to the eradication and non-eradication groups, respectively. Figure 1 depicts the flow diagram of enrolled patients. Among them, 69.1% were males, with a mean (standard deviation) age of 61.6 (9.7 years). The lesions showed elevated morphology in >50% of the total patients (*n* = 338, 50.2%), and the mean (standard deviation) size of the lesion was 11.7 mm (7.8 mm). The lesion was in the lower third of the stomach in 42.2% (*n* = 284) of the total patients, and 15.8% (*n* = 106) had high-grade dysplasia. Less than 10% of the total patients had a familial history of gastric cancer (*n* = 63, 9.4%), and less than 50% of the total patients were smokers (*n* = 251, 37.3%). Patients with 1 point of ASA physical status score were more than 80% of the total patients (*n* = 563, 83.7%), and the proportion of alcohol drinkers was also revealed to be similar to that of smokers (*n* = 250, 37.1%). A comparison of the baseline characteristics of the eradication and non-eradication groups revealed that most investigated variables, including clinical parameters and pathologic information, did not differ between the groups. However, smoking history and tumor gross morphology differed between the two groups (*p* for all < 0.05). In the eradication group, less than 30% were smokers (*n* = 48, 29.4%). However, the proportion of smokers in the non-eradication group was up to 40% (*n* = 203, 39.8%). In the eradication group, the rates of elevated and flat morphologies were similar. In contrast, more than half of the non-eradication group had superficial elevated morphology (*n* = 276, 54.1%). In addition, the eradication group had a 1.60 times higher proportion of tumors with depressed morphology than the non-eradication group (25.2% vs. 15.3%, *p* = 0.001). Table 1 details the baseline characteristics of the two groups.

### 3.2. Metachronous Gastric Neoplasm after ESD and Risk Factors Associated with Metachronous Gastric Neoplasm

The mean follow-up period was 25 (interquartile range (IQR): 18–35 months) and 39 months (IQR: 27–57 months) in the eradication and non-eradication groups, respectively. In the total patient population, there were 28 cases (4.2%) of metachronous gastric neoplasm after ESD. The incidence rate of metachronous gastric neoplasm did not differ significantly between the two groups (6 (3.7%) vs. 22 (4.3%), *p* = 0.825). In the non-eradication group, all metachronous gastric adenoma cases had low-grade dysplasia (*n* = 13). Metachronous gastric cancer was observed in three (1.8%) and nine (1.8%) patients of the eradication and non-eradication groups, respectively, and the incidence rate of metachronous gastric cancer was similar between the two groups (Table 2).

**Figure 2:**
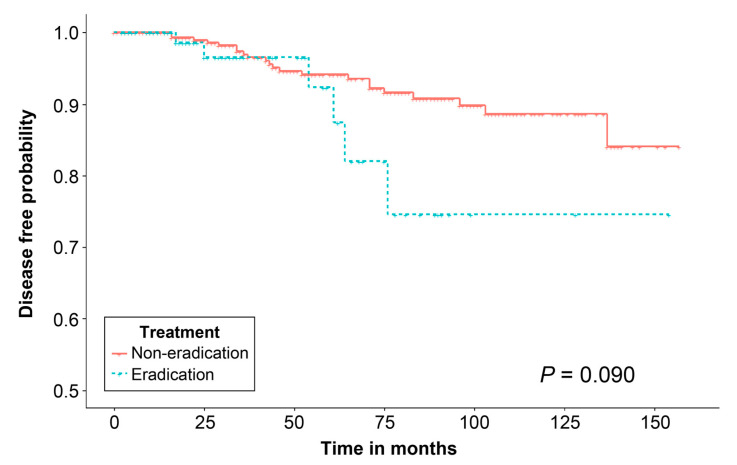
Kaplan–Meier analysis of the disease-free probability of metachronous gastric neoplasm after endoscopic submucosal dissection for gastric adenoma.

In our analysis of variables that could increase the risk of metachronous gastric neoplasm, no risk factor for metachronous gastric neoplasm was identified. The success of *H. pylori* eradication treatment after ESD for gastric adenoma resection was not a risk factor for metachronous gastric neoplasm (HR: 0.848, 95% CI: 0.338–2.128, *p* = 0.725) (Table 3). Kaplan–Meier analysis demonstrated that the disease-free probability did not differ significantly between the two groups, regardless of whether patients received eradication treatment after ESD (*p* = 0.090) (Figure 2).

### 3.3. Effect of H. pylori Eradication Treatment on Propensity Score-Matched Population

**Figure 3:**
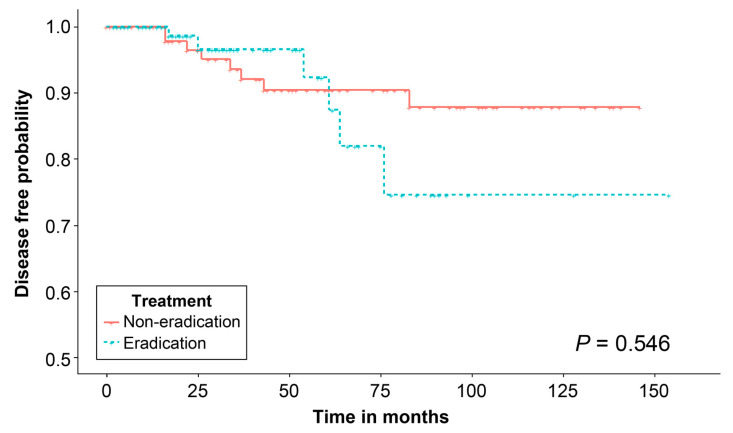
Kaplan–Meier analysis of the disease-free probability of metachronous gastric neoplasm after endoscopic submucosal dissection for gastric adenoma in the matched population.

This study did not identify any risk factor for metachronous gastric neoplasm; however, a 1:1 propensity score matching analysis was conducted to adjust for variables previously identified as risk factors for metachronous gastric neoplasm [19]. The variables included age, sex, pathology, chronic atrophic gastritis, intestinal metaplasia, lesion size, tumor location, gross morphology, familial history of gastric cancer, smoking, and ASA physical status score. Differences in baseline characteristics were eliminated after matching (Table 4), and the distribution of propensity scores is described in Supplementary Figure S1. However, Kaplan–Meier analysis of the matched population demonstrated that the disease-free probability did not differ significantly between the two groups, regardless of whether patients received eradication treatment after ESD (*p* = 0.546) (Figure 3).

## 4. Discussion

Eliminating risk factors is the most basic strategy for preventing gastric cancer. As *H. pylori* is a well-known risk factor in gastric cancer [25], the importance of diagnosing and treating *H. pylori* infection is growing. In different randomized studies, *H. pylori* eradication treatment reduced the incidence of metachronous cancer after endoscopic resection of early gastric cancer [18,26,27]. However, evidence of the role of *H. pylori* eradication treatment in gastric adenoma, a precancerous lesion, is lacking. Herein, we used single-center data accumulated over 15 years to investigate the effects of *H. pylori* eradication treatment on metachronous lesions after gastric adenoma resection. Local recurrence and synchronous lesions that were observed within a year after ESD were excluded. Only cases where lesions resected through ESD could undergo full histopathologic evaluation were enrolled for analysis. Therefore, our findings revealed that *H. pylori* eradication treatment after gastric adenoma resection did not affect the incidence of metachronous gastric neoplasm. Intriguingly, the replication of these results, even after propensity score matching, confirmed that *H. pylori* eradication could not influence metachronous gastric neoplasm after ESD.

According to Correa’s multi-stage cascade of gastric oncogenesis, prolonged mucosal inflammation causes changes in atrophy and intestinal metaplasia [28]. Further progression leads to low- or high-grade intraepithelial neoplasia. Gastric adenoma, classified as low- or high-grade intraepithelial neoplasia in the Revised Vienna classification [23], is an advanced precancerous lesion that may progress into invasive gastric cancer [28]. Therefore, intraepithelial neoplasm, including gastric adenoma, is occasionally considered a “bridge” connecting atrophy/intestinal metaplasia and gastric cancer [29]. In this stepwise progression, *H. pylori* is a risk factor in gastric cancer. Thus, eradication treatment was reported to lower incidence and mortality in patients with gastric cancer [30,31,32,33]. Hence, the International Agency for Research on Cancer considered *H. pylori* a human carcinogen [1]. Therefore, endoscopic resection is recommended for gastric adenoma, a precancerous lesion [15]. The necessity for *H. pylori* eradication treatment is debatable in conditions with or without atrophy/intestinal metaplasia after gastric adenoma resection. Resecting gastric adenoma lesions does not change the status of the gastric mucosa. In the stepwise progression from normal to gastric cancer, gastric adenoma is an intermediate neoplasm. Thus, resecting the lesion is not a fundamental treatment for gastric mucosa, and these theoretical bases suggest that *H. pylori* eradication treatment must be performed after resecting gastric adenoma.

However, in the “point of no return” theory, where gastric mucosa with atrophy/intestinal metaplasia is present in the stepwise progression, *H. pylori* eradication treatment cannot reverse the progressive histological changes [12]. According to two published meta-analysis studies, *H. pylori* eradication treatment reduced the risk of future gastric cancer in patients without precancerous lesions or with atrophy. However, *H. pylori* eradication did not reduce the risk of gastric cancer in patients with intestinal metaplasia or gastric adenoma [34,35]. Moreover, the severity of atrophic gastritis influenced the effects of *H. pylori* eradication treatment. *H. pylori* eradication treatment was more effective in preventing gastric cancer in patients with mild atrophy than in patients with extensive atrophy [36]. These findings support the “point of no return” theory. Additionally, in a randomized controlled trial, molecular markers related to carcinogenesis, such as microsatellite instability, human mutL homolog 1, cyclin-dependent kinase inhibitor 2A, methylation of adenomatous polyposis coli genes, and reactivity of monoclonal antibody Das-1, were analyzed in gastric mucosa with intestinal metaplasia. The participants were divided into eradication and non-eradication groups for re-analysis of molecular markers a year later; however, the molecular markers did not differ significantly between the two groups, suggesting that *H. pylori* eradication treatment did not improve molecular changes related to carcinogenesis [37]. This is consistent with our findings, where successful eradication treatment did not affect the incidence of gastric cancer or metachronous gastric neoplasm during the follow-up period.

In contrast, in a large cohort study of the general population conducted in China, *H. pylori* eradication treatment reduced the risk of gastric cancer in patients with intestinal metaplasia or gastric adenoma [13]. In that study, the patients were followed up for 15 years, demonstrating the long-term effects of *H. pylori* eradication treatment. In a study that analyzed the effects of *H. pylori* eradication treatment on the incidence of metachronous gastric cancer after endoscopic resection of gastric cancer, the eradication group showed histological improvement of atrophy/metaplasia after 3 years of follow-up [18]. The effects of eradication treatment were not observed after 3 years of follow-up [38], but after 6 years of follow-up, the eradication group had a significantly decreased incidence of metachronous gastric cancer [27]. This improvement was observed after 5 years of eradication. Therefore, the follow-up period in previous studies supporting the “point of no return” theory and in our study may have been insufficient. The results may change after several years of follow-up.

In our study, a synchronous lesion was defined as a newly observed lesion at a site other than the ESD site within a year of the procedure. Along with cases that recurred at the ESD site (defined as residual disease), synchronous lesions were excluded from the final analysis. This was because cases of newly observed lesions within a year may be newly developed cancer or possibly missed cancer. Previous studies that evaluated the effects of *H. pylori* eradication treatment after gastric adenoma resection included cases of newly observed lesions within a year [21,26,38]. In those studies, the overall difference in incidence of newly observed lesions according to eradication success was attributed to the difference in newly observed lesions within the first-year post-endoscopic resection. Therefore, we excluded these patients as they would have interfered with our analysis of the effects of *H. pylori* eradication on the incidence of metachronous gastric neoplasm. Additionally, unlike previous studies that compared the incidence of metachronous gastric neoplasm by comparing eradication and non-eradication groups without confirming the success of eradication treatment [27], we only selected patients whose eradication treatment were successful. In South Korea, the eradication success rate of standard triple therapy ranges between 68.4% and 83.0%, suggesting that the failure rate of eradication could be as high as 31.6% [39,40]. Herein, we only enrolled patients who had successful eradication treatment, excluding variables affecting metachronous gastric neoplasm due to eradication failure and strengthening our results.

This study had some limitations. First, this study was conducted in a single center. However, the pathological evaluation and detection of *H. pylori* after ESD were conducted under the same conditions, limiting the effects of various confounding variables. Second, this study was retrospective; hence, various confounding factors might have interfered with interpreting the results. Therefore, to minimize baseline differences between the eradication and non-eradication groups, we removed known risk factors for gastric neoplasm development through matching. Nevertheless, factors such as the degree of gastric atrophy, gastric hypoacidity, and secondary cancer risk should be analyzed as factors that could affect eradication treatment. Given the retrospective design of this study, it was not possible to evaluate the above factors. Third, this study did not evaluate *H. pylori* re-infection following eradication treatment. This study only analyzed patients who had successful eradication treatment to clarify the effects after eradication treatment. However, some patients may have been reinfected with *H. Pylori* after successful eradication treatment. *H. pylori* re-infection was not evaluated; however, the annual rate of *H. Pylori* infection in South Korea is approximately 3.5%, which would have had minimal effects on the study results [41]. Finally, several patients had a relatively short follow-up period. This issue may have led to the misinterpretation of the study results on the effect of *H. pylori* eradication on metachronous gastric neoplasm.

## 5. Conclusions

In conclusion, our results demonstrate that the success of *H. pylori* eradication treatment after ESD with curative resection of gastric adenoma did not show difference in the incidence of metachronous gastric neoplasm. In addition, our findings were reported after removing baseline differences between the eradication and non-eradication groups through propensity score matching analysis. However, future studies should include a longer follow-up period, because eradication treatment eliminates *H. pylori*, a major risk factor in gastric cancer, and the effects of eradication treatment may appear after a long period of time.

## Figures and Tables

**Table 1:** Baseline characteristics of the study population.

Clinical Parameter	Total (*n* = 673)	Eradication (*n* = 163)	Non-Eradication (*n* = 510)	*p*-Value
Age, years, mean ± SD	61.6 ± 9.7	60.9 ± 9.2	61.9 ± 9.8	0.284
Male, *n* (%)	465 (69.1)	110 (67.5)	355 (69.6)	0.627
Familial history of gastric cancer, *n* (%)				0.279
Yes	63 (9.4)	19 (11.7)	44 (8.6)	
No	610 (90.6)	144 (88.3)	466 (91.4)	
Smoking, *n* (%)				0.020
Yes	251 (37.3)	48 (29.4)	203 (39.8)	
No	422 (62.7)	115 (70.6)	307 (60.2)	
Alcohol drinker, *n* (%)				0.352
Yes	250 (37.1)	66 (40.5)	184 (36.1)	
No	423 (62.9)	97 (59.5)	326 (63.9)	
ASA physical status, *n* (%)				0.626
ASA 1	563 (83.7)	134 (82.2)	429 (84.1)	
ASA 2	110 (16.3)	29 (17.8)	81 (15.9)	
Lesion size, mm, mean ± SD	11.7 ± 7.8	12.1 ± 7.6	11.6 ± 7.9	0.446
Gross morphology type, *n* (%)				0.001
Elevated	338 (50.2)	62 (38.0)	276 (54.1)	
Flat	216 (32.1)	60 (36.8)	156 (30.6)	
Depressed	119 (17.7)	41 (25.2)	78 (15.3)	
Tumor Location, *n* (%)				0.486
Upper 1/3	96 (14.3)	19 (11.7)	77 (15.1)	
Middle 1/3	293 (43.5)	76 (46.6)	217 (42.5)	
Lower 1/3	284 (42.2)	68 (51.0)	216 (42.4)	
Pathology, *n* (%)				0.035
Low-grade dysplasia	567 (84.2)	146 (90.8)	421 (82.5)	
High-grade dysplasia	106 (15.8)	17 (10.4)	89 (17.5)	
Atrophic gastritis, *n* (%)				0.113
Yes	583 (86.6)	135 (82.8)	448 (87.8)	
No	90 (13.4)	28 (17.2)	62 (12.2)	
Intestinal metaplasia, *n* (%)				0.069
Yes	579 (86.0)	133 (81.6)	446 (87.5)	
No	94 (14.0)	30 (18.4)	64 (12.5)	

Abbreviations: ASA, American Society of Anesthesiologists; SD, standard deviation.

**Table 2:** Incidence and characteristics of metachronous gastric neoplasm after endoscopic submucosal dissection between the two groups.

Variable	Eradication (*n* = 163)	Non-Eradication (*n* = 510)	*p*-Value
Metachronous gastric neoplasm, *n* (%)	6 (3.7)	22 (4.3)	0.825
Adenoma	3 (1.8)	13 (2.5)	0.773
Pathology			0.188
Low-grade dysplasia	2 (66.7)	13 (100)	
High-grade dysplasia	1 (33.3)	0 (0)	
Cancer	3 (1.8)	9 (1.8)	1.000
Pathology			1.000
Differentiated	2 (66.7)	7 (77.8)	
Undifferentiated	1 (33.3)	2 (22.2)	

*p*-values were based on Fisher’s exact test.

**Table 3:** Cox proportional hazard model for incidence risk of metachronous gastric neoplasm after endoscopic submucosal dissection.

	Univariable Analysis	
	HR (95% CI)	*p*-Value
Age, years	1.019 (0.979–1.059)	0.357
Sex		
Male	Ref.	
Female	0.890 (0.385–2.055)	0.785
Age group		
50–54	0.310 (0.062–1.556)	0.155
55–59	1.944 (0.656–5.763)	0.231
60–64	1.040 (0.310–3.487)	0.949
65–69	2.045 (0.667–6.274)	0.211
≥70	Ref.	
Smoker	1.722 (0.807–3.673)	0.160
Alcohol drinker	0.938 (0.426–2.064)	0.873
ASA physical status		
ASA 1	Ref.	
ASA 2	1.118 (0.416–3.007)	0.825
Success for *Helicobacter pylori* infection		
No	Ref.	
Yes	0.848 (0.338–2.128)	0.725
Lesion size, mm	1.016 (0.972–1.062)	0.492
Gross morphology		
Elevated or flat	Ref.	
Depressed	0.547 (0.162–1.843)	0.331
Tumor location		
Lower third	Ref.	
Middle third	0.920 (0.328–2.583)	0.875
Upper third	0.410 (0.166–1.012)	0.053
Pathology		
Low-grade dysplasia	Ref.	
High-grade dysplasia	0.631 (0.187–2.130)	0.459
Atrophy	1.299 (0.384–4.395)	0.674
Intestinal metaplasia	1.369 (0.405–4.627)	0.613

Abbreviations: ASA, American Society of Anesthesiologists; CI, confidence interval; HR, hazard ratio.

**Table 4:** Baseline characteristics of the study population after propensity score matching.

Clinical Parameter	Total (*n* = 326)	Eradication (*n* = 163)	Non-Eradication (*n* = 163)	*p*-Value	SMD
Age, years, mean ± SD	60.9 ± 9.4	60.9 ± 9.2	60.8 ± 9.6	0.921	0.011
Male, *n* (%)	215 (66.0)	110 (67.5)	105 (64.4)	0.640	0.065
Familial history of gastric cancer, *n* (%)				0.513	0.091
Yes	43 (13.2)	19 (11.7)	24 (14.7)		
No	283 (86.8)	144 (88.3)	139 (85.3)		
Smoking, *n* (%)				0.810	0.040
Yes	99 (30.4)	48 (29.4)	51 (31.3)		
No	227 (69.6)	115 (70.6)	112 (68.7)		
Alcohol drinker, *n* (%)				0.570	0.045
Yes	126 (38.7)	66 (40.5)	60 (36.8)		
No	200 (61.3)	97 (59.5)	103 (63.2)		
ASA physical status, *n* (%)				0.883	0.033
ASA 1	270 (82.8)	134 (82.2)	136 (83.4)		
ASA 2	56 (17.2)	29 (17.8)	27 (16.6)		
Lesion size, mm, mean ± SD	12.0 ± 8.2	12.1 ± 7.6	12.0 ± 8.8	0.893	0.015
Gross morphology type, *n* (%)				1.000	0.026
Elevated	125 (38.3)	62 (38.0)	63 (38.7)		
Flat	118 (36.2)	60 (36.8)	58 (35.6)		
Depressed	83 (25.5)	41 (25.2)	42 (25.8)		
Tumor Location, *n* (%)				0.310	0.125
Upper 1/3	33 (10.1)	19 (11.7)	14 (8.6)		
Middle 1/3	149 (45.7)	76 (46.6)	73 (44.8)		
Lower 1/3	144 (44.2)	68 (51.0)	76 (46.6)		
Pathology, *n* (%)				0.853	0.041
Low-grade dysplasia	294 (90.2)	146 (90.8)	148 (89.6)		
High-grade dysplasia	32 (9.8)	17 (10.4)	15 (9.2)		
Atrophic gastritis, *n* (%)				1.000	0.016
Yes	269 (82.5)	135 (82.8)	134 (82.2)		
No	57 (17.5)	28 (17.2)	29 (17.8)		
Intestinal metaplasia, *n* (%)				1.000	<0.001
Yes	266 (81.6)	133 (81.6)	133 (81.6)		
No	60 (18.4)	30 (18.4)	30 (18.4)		

Abbreviations: ASA, American Society of Anesthesiologists; SD, standard deviation; SMD, standardized mean difference.

## Data Availability

The datasets analyzed and/or used in this study are available from the corresponding author upon reasonable request.

## Supplementary Materials

The following supporting information can be downloaded at: https://www.mdpi.com/article/10.3390/jcm12041512/s1, Figure S1: Plot of absolute standardized differences in baseline characteristics before and after propensity score matching.
