# Supplementary figures and images for: Effect of Helicobacter pylori Eradication Treatment on Metachronous Gastric Neoplasm Prevention Following Endoscopic Submucosal Dissection for Gastric Adenoma
Source: J Clin Med. 2023 Feb 14;12(4):1512. doi: 10.3390/jcm12041512 (PMC9962017; doi:10.3390/jcm12041512)

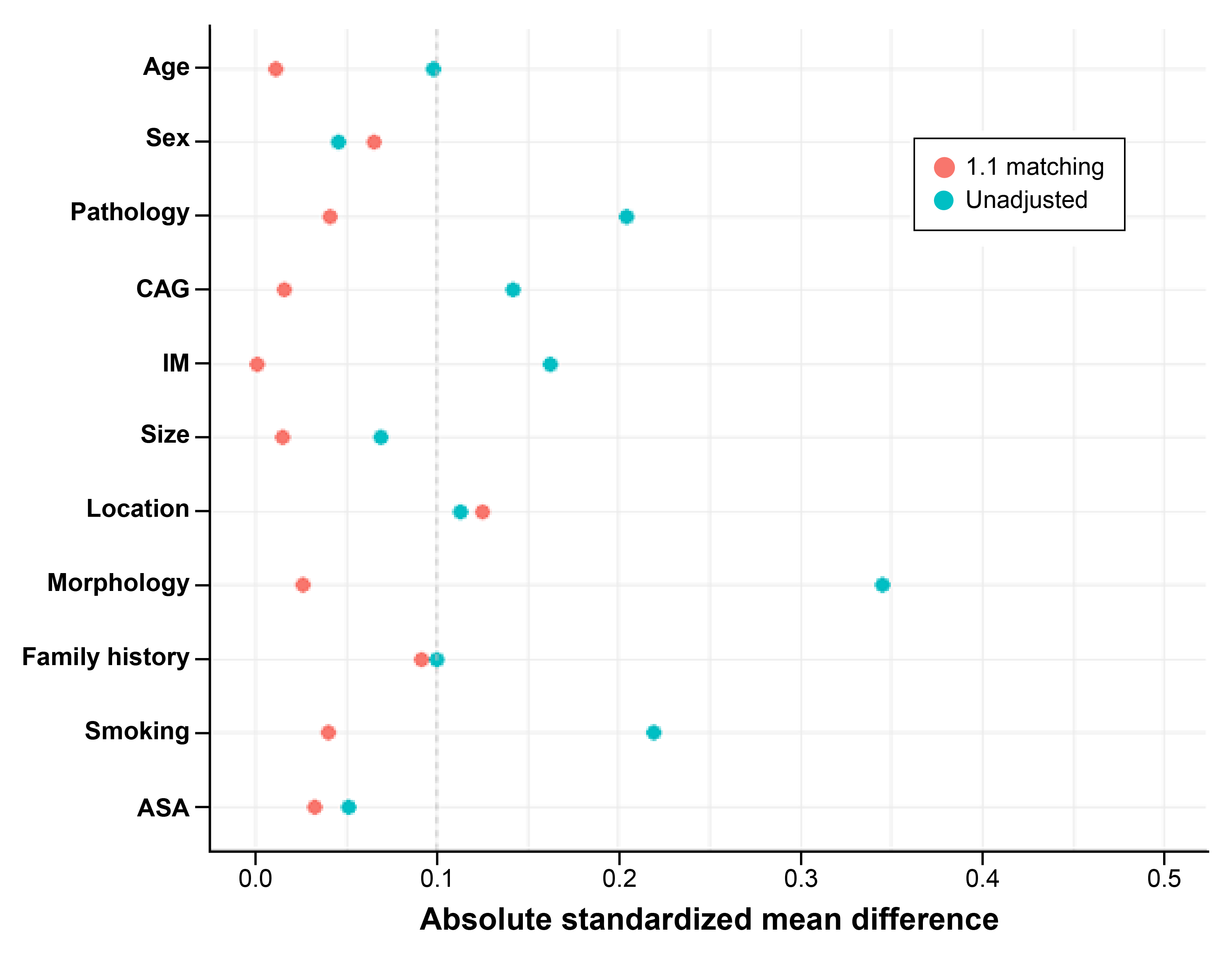

Supplement: Supplementary file 1 [file jcm-12-01512-s001.zip › jcm-2178355-supplementary Figure S1.tif]
